# Creatine Supplementation to Improve Sarcopenia in Chronic Liver Disease: Facts and Perspectives

**DOI:** 10.3390/nu15040863

**Published:** 2023-02-08

**Authors:** Riccardo Casciola, Laura Leoni, Biagio Cuffari, Maddalena Pecchini, Renata Menozzi, Antonio Colecchia, Federico Ravaioli

**Affiliations:** 1 Gastroenterology Unit, Department of Specialistic Medicines, University of Modena & Reggio Emilia, University Hospital of Modena and Reggio Emilia, Largo del Pozzo 71, 41125 Modena, Italy; 2 Division of Metabolic Diseases and Clinical Nutrition, Department of Specialistic Medicines, University Hospital of Modena and Reggio Emilia, Largo del Pozzo 71, 41125 Modena, Italy; 3 Department of Medical and Surgical Sciences, University of Bologna, 40138 Bologna, Italy

**Keywords:** chronic liver disease, nutrition, ACLD, advanced liver diseases, portal hypertension, cirrhosis, creatine, supplementation, sarcopenia, muscle, fatigue

## Abstract

Creatine supplementation has been one of the most studied and useful ergogenic nutritional support for athletes to improve performance, strength, and muscular mass. Over time creatine has shown beneficial effects in several human disease conditions. This review aims to summarise the current evidence for creatine supplementation in advanced chronic liver disease and its complications, primarily in sarcopenic cirrhotic patients, because this condition is known to be associated with poor prognosis and outcomes. Although creatine supplementation in chronic liver disease seems to be barely investigated and not studied in human patients, its potential efficacy on chronic liver disease is indirectly highlighted in animal models of non-alcoholic fatty liver disease, bringing beneficial effects in the fatty liver. Similarly, encephalopathy and fatigue seem to have beneficial effects. Creatine supplementation has demonstrated effects in sarcopenia in the elderly with and without resistance training suggesting a potential role in improving this condition in patients with advanced chronic liver disease. Creatine supplementation could address several critical points of chronic liver disease and its complications. Further studies are needed to support the clinical burden of this hypothesis.

## 1. Introduction

Creatine (Cr) (*N*-aminoiminomethyl-*N*-methyl glycine) is a compound spontaneously occurring in nature [1,2].

Creatine plays an important energy and anticatabolic function by increasing phosphocreatine (PCr) levels and permitting a rapid regeneration of ATP after a burst of ATP utilisation in human tissues, which continuously require a replacement of creatine stores. Creatine’s beneficial effect increases the reservoir and capacity of anaerobic energy by decreasing protein catabolism and increasing muscle mass and physical performance [1].

For this reason, creatine supplementation has been one of the most studied and useful ergogenic nutritional support for athletes [2]. While these known creatine-positive effects benefit athletes, creatine could also have a clinical and therapeutic application in human diseases. Over the past decade, evidence on the role of creatine supplementation for sarcopenia in chronic disease has been investigated [1,3,4,5]. Since endogenous creatine synthesis is primarily in the liver, a presumed role of creatine in liver disease is expected [3]. In this review article, we aimed to summarise the current evidence for creatine supplementation in advanced chronic liver disease and primarily in sarcopenic cirrhotic patients who would potentially benefit from creatine supplementation.

## 2. Data Sources and Searches

We searched English language publications on MEDLINE, Ovid, In-Process, Cochrane Library, EMBASE and PubMed up to November 2022. Literature searches were performed using the following keywords: Creatine, *N*-aminoiminomethyl-*N*-methyl glycine, creatine supplementation, creatine safety, liver diseases, chronic liver disease, advanced chronic liver disease, chronic disease, cirrhosis, sarcopenia, sarcopenic, muscle depletion, muscle mass.

## 3. Creatine: Instruction for Use

### 3.1. Definition

Creatine is a guanidino compound naturally present in nature and mainly synthesised in the liver, kidneys, and pancreas [6]. It is known for its role in the intracellular storage of metabolic energy. Creatine is converted to phosphocreatine (PCr) by creatine kinase (CK), which catalyses the reversible transference of a phosphoryl group, allowing a rapid phosphate high energy bond exchange operated to generate adenosine triphosphate (ATP). This form of energy storage is particularly important in tissues with high and rapid energetic needs, such as muscles and the brain [1,6,7].

Creatine is present naturally in food, particularly in meat and fish; humans on a western diet get about one-half of their creatine from the diet and one-half by synthesis. From diet intake, the daily nutritional requirement averages 1–2 g of creatine [8]. The importance of endogenous synthesis of creatine is related to the physiological spontaneous and irreversible conversion to creatinine and, subsequently, urine loss. For example, in a 70-kg male in normal conditions, there is a loss of about 1–2 g of creatine [1,3,4,8,9]. 

### 3.2. Biosynthesis and Metabolism

**Figure 1:**
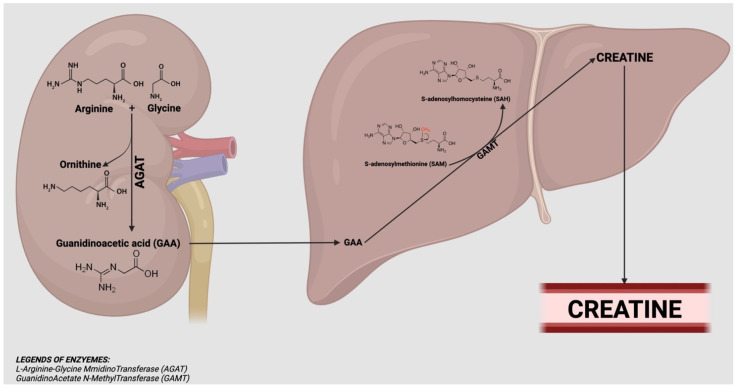
Creatine biosynthesis: GAA is synthesised in the kidney from arginine and glycine in a reaction catalysed by AGAT. Then GAA is released into the blood circulation and transported to the liver, transforming it into creatine with the contribution of GAMT and SAM. Finally, creatine is distributed via the bloodstream mainly to muscles and the brain.

Figure 1 summarizes the process of creatine biosynthesis. This pathway needs two steps that require three amino acids (arginine, glycine and methionine) and two enzymes, L-arginine-glycine amidinotransferase (AGAT) and guanidinoacetate N-methyltransferase (GAMT). AGAT is known to be active in the kidney and GAMT in the liver of various animal species. In the first step, the amidino group from arginine is transferred to the amino group of glycine, yielding ornithine and guanidinoacetic acid (GAA), a reaction catalysed by AGAT. In the second step, GAA is methylated by GAMT on the original glycine nitrogen, using S-adenosylmethionine (SAM) as the methyl donor to form S-adenosylhomocysteine (SAH) and creatine. Interestingly, it was supposed that in humans, GAA must be synthesised in tissues other than the kidney because it represents only 20% of the daily loss of creatinine [3,10]. In line with this hypothesis, it was supposed that the entire creatine synthetic pathway could be formed in the liver, and the arginine could be acted upon by either arginase to form urea or by AGAT to form GAA [3,11]. Therefore, in literature, an AGAT mRNA expression in the human liver is reported [3,12]. Other support for this hypothesis came from immunohistochemical evidence of AGAT in the cytosol of rat hepatocytes [3,13]. Finally, creatine released from the liver into circulation can reach target tissues such as the muscles [14].

In this background, the key role of creatine in humans organism is underlined, not only by the primary and well-known part in the biochemical mechanism of cellular energy but mostly by the evidence of a series of signs and symptoms such as hypotonia, muscle weakness, speech impairment, brain atrophy, mental retardation, autism that characterised creatine deficiency syndrome that is led from genetical defects in the synthesis (AGAT, GAMT or creatine transporter) [15,16].

### 3.3. Dosage, Formulation and Method of Administration

The most commonly recommended dose of creatine supplementation range from 3 to 5 g/day or 0.1 g/Kg/day, with a common use in athletes of 5 g/day, although it can reach 20–25 g/day (or 0.3 g/Kg/day) in a loading phase [17,18]. To date, the current evidence has demonstrated how a loading phase is not necessary since a lower dosage of creatine (3–5 g/day) can increase intramuscular creatine stores leading to the beneficial effects on muscles and performance seen with a higher dosage, especially if the supplementation period is over 30 days [18]. Notably, it is suggested to split the creatine dosage into smaller doses (usually 5 g for four times/day) throughout the day. The reasons behind the fractionation of the dosage are several and mainly related to the major solubility of the creatine monohydrate [18] and less risk of gastrointestinal distress (i.e., diarrhoea) [19]. Because of its ampholytic property, creatine shows the best solubility at 14 g/L and 20 °C with a neutral PH of 7; for this reason, mixing it in high-temperature solution increases solubility, although this trick does not influence tissue uptake [2]. In a healthy subject, correct creatine supplementation induces the saturation of muscular storage in about 28 days. Return to the basal status occurs about 4–6 weeks after the discontinuation [2,20,21]. The preferential way of administration of creatine is with food, especially with a carbohydrate meal, because creatine transport into blood circulation and muscles is facilitated by insulin action [21]: this depends on the insulin capacity of facilitates sodium-dependent creatine transport [22].

For what concern formulation, monohydrate creatine powder (available in tablets or capsules) has been the most studied and chosen form of creatine because of its evidence-based efficacy. Over the years, other creatine formulations have come out on the global market, focusing on better solubility than the monohydrate form. However, published papers have yet to show any major effectiveness of those other forms compared to the monohydrate. Monohydrates have the best price-effectiveness ratio and are currently the best [18,21].

### 3.4. Creatine Safety

Regarding the safety of creatine supplementation, several studies evaluating the hypothetical toxicity of creatine supplementation (at the recommended dose) in humans have not found any evidence of side effects for the kidney and liver [1,3,9,23]. In particular, a multicenter study of more than 1500 patients demonstrated no alterations in kidney and liver parameters associated with creatine supplementation [24]. 

There are contradictory data about the oxidant/antioxidant effect of creatine. Several studies have shown a decreasing marker of oxidative stress in animal models of neurodegenerative disease [3,25,26,27]. Indeed, in the liver of rats subjected to exercise, it has been shown that creatine supplementation could improve the activity of some antioxidant enzymes like glutathione peroxidase and catalase [3,28] and produce antioxidant effects by scavenging reactive oxygen species [3,29]. Another recent study on rats demonstrated how creatine supplementation protected against doxorubicin-induced liver toxicity by attenuating inflammation, oxidative stress, cellular senescence and fibrosis [30]. On the other hand, there are studies in rats underlying potential negative effects of creatine supplementation, such as increased oxidative stress and potentially forming carcinogenic compounds in vitro [3] and exacerbating in mice ethanol-induced hepatic damage by interfering in ethanol metabolism and causing ethanol-induced inflammation and oxidative stress [31]. 

The only directly demonstrated side effect of Cr supplementation is weight gain due to the increase in total intracellular body water without altering fluid distribution [23].

The general conclusion on the safety of creatine supplementation on health is that there is no clear evidence to affirm that creatine supplementation may affect kidney and liver function in healthy subjects [17,18,24], and in any case, the major risk of creatine supplementation is probably linked to the purity of commercially available creatine [3,32].

## 4. Creatine: Role in Health and Disease

### 4.1. Creatine in Healthy Young and Elderly with and without Resistance Training

For the ergogenic property of creatine, it has been the most widely studied supplementation and useful nutritional support for athletes [2]. Creatine supplementation has been broadly studied in athletes or people who underwent physical training or sport and widely demonstrating to improve muscle mass, strength, and performance in over 1000 studies on young and middle-aged subjects [17,33]. Even in elderly patients, a recent systematic review [34] and meta-analysis [35] have concluded that creatine is a safe supplement associated with resistance training to increase muscular mass. Moreover, in those ageing people who do not perform physical training, creatine supplementation can improve quality of life by delaying muscular atrophy and improving endurance and strength, allowing to preserve muscular performance for everyday tasks [9,36]. Data showing a beneficial effect from creatine supplementation on ageing muscle without concomitant resistance training are biased. Five studies have shown positive effects, four studies have shown no effect, and one has shown mixed effects [37,38,39,40,41,42,43,44,45,46]. Stout et al. [37] found a significant improvement in maximal hand grip strength and physical work capacity at fatigue threshold in older adults (15 men and women, 74.5 ± 6.4 years) who took the creatine monohydrate supplementation (20 g/day for 7 days and then 10 g/day for 7 days) versus placebo. Rawson et al. [38] demonstrated that 1 month of creatine monohydrate supplementation (20 g/day for 10 days and then 4 g/day for 20 days) reduced lower body muscle fatigue in older men but did not increase body composition or strength (20 subjects aged 60 to 82 years). Gotshalk et al. [39,40] described improved strength and function in 20 men, aged 59 to 73 years and 30 women, aged 58 to 71 years, who took creatine monohydrate supplementation (0.3 g/kg/day) for 1 week: particularly elderly women [40] showed a significant increase in the bench press (1.7 +/− 0.4 kg), leg press (5.2 +/− 1.8 kg), body mass (0.49 +/− 0.04 kg) and lean mass (0.52 +/− 0.05).

### 4.2. Creatine as a New Treatment for Improving Health and Quality of Life for Patients with Chronic Kidney Disease

Chronic kidney disease (CKD) is a condition that can reduce muscle mass and neurological functioning associated with a poor quality of life. A recent review [17] supports that creatine supplementation can improve CKD patients’ muscular, bone, metabolic, immune, central and peripheral nervous systems. Authors showed wide and convincing evidence to ensure the safety of creatine supplementation even in CDK patients with a lower serum concentration than healthy controls [47], proving an impaired creatine synthesis metabolism. In those patients to whom creatine was administered, a single effect of reduced muscle cramps and increased quality of life was shown. Finally, an intradialytic infusion of creatine has been demonstrated to bypass ingestion and compliance limits in CKD patients. 

### 4.3. Creatine in Other Disease Conditions

Creatine has also demonstrated a role in other diseases, such as diabetes, osteoporosis, cancer, and cardiovascular and neurological diseases [1,3,4,5]. In addition to the aforementioned beneficial effects of creatine, a series of interesting direct and indirect cellular effects have also been demonstrated, such as the protection of tissues from ischemic and oxidative insults, the reduction of inflammatory markers, the antiapoptotic [48,49], antidiabetic [50,51], lipid-lowering role [52,53], and modulator of the immune and the intestinal system [54,55].

Finally, a series of studies showed how creatine supplementation could improve beyond the more intuitive physical fatigue [33,56], the cognitive one in humans and be beneficial for brain performance [56,57,58,59].

## 5. Creatine and Liver

The liver is a key organ in the endogenous synthesis of creatine thus, in patients with abnormal liver function, creatine may be produced at a lower level contributing to subsequent manifestations in liver disease such as sarcopenia, fatigue, and encephalopathy.

### 5.1. Creatine and Chronic Liver Diseases

In our knowledge, only in rat models of NAFLD/NASH, the benefit of creatine supplementation has been demonstrated by decreasing homocysteine (Hcy) production in the liver, diminishing fat accumulation and bringing beneficial effects in the fatty liver [3]. Moreover, creatine supplementation can also prevent fatty liver, as shown in two studies [60,61] performed in rats fed with a high-fat diet and choline-deficient diet plus creatine compared to those without creatine. In humans, a study performed on 34 subjects showed how creatine monohydrate supplementation for 56 days (20 g/day for 5 days and then at 5 gr/day for 51 days) might significantly reduce blood lipids at 4 and 8 weeks (significant reductions in total plasma cholesterol by 6% and 5% respectively, triacylglycerols and very-low-density lipoprotein-C by 23% and 22% respectively) [53]. 

Fat accumulation and NAFLD/NASH progression are strictly associated with the shortage of methionine metabolism in the liver, leading to diminished availability of SAM, an elevation in Hcy levels and oxidative stress generation. Normally creatine biosynthesis uses SAM stores and also produces Hcy in the liver. In line, creatine supplementation is known to decrease the consumption of SAM and to reduce the Hcy production in the liver, leading to a decrease of triglycerides and fat synthesis and subsequent liver accumulation [52]. Creatine supplementation is related to reduced endogenous creatine formation, down-regulation of renal AGAT activity, and impairment of Hcy production [3,61]. 

Hcy reduction has been considered one of the mechanisms of creatine beneficial properties because high levels of Hcy increase the risk of a series of diseases, including renal and liver ones, in animal models [3,62]. Moreover, it was demonstrated in rats fed a high-fat diet that dietary creatine supplementation can positively alter hepatic lipid metabolism by intensifying lipoprotein secretion and oxidation [63].

On the other side, the development of alcoholic steatosis seems not to be prevented by creatine supplementation [64]. Consequently, creatine seems not to have a beneficial role in alcohol-damaged liver, and alcohol negatively alters creatine synthesis [65]. Hence, a hypothetical supplementation is useless in this case and exacerbates ethanol-induced hepatic damage in mice [31].

### 5.2. Creatine and Hyperammonaemia in Advanced Chronic Liver Disease

Creatine supplementation has demonstrated a role even in hyperammonemia, a condition linked to hepatic encephalopathy, a complication of acute and chronic liver failure. In fact, creatine, phosphocreatine, and creatine kinase are important in maintaining cellular energy homeostasis that would prevent the neurodegenerative process. Hepatic encephalopathy is linked with increased ammonia levels in the blood and the brain with decreased brain energy and neuronal activity [66]. Therefore, an interesting study showed how a mouse model of late-stage alcoholic liver fibrosis was studied to investigate neurochemical alterations in the thalamus and their link to behavioural changes, highlighting that thalamus levels of creatine were significantly lower and strictly correlated with alcohol-induced behavioural changes [67].

Studies have shown that creatine supplementation could supply brain energy, leading to neuroprotective effects against hyperammonemia-induced encephalopathy [66,68]. Curiously, the lack of brain energy caused by hyperammonemia seems to be able to increase creatine uptake in brain endothelial cells [3,66,68,69].

Notably, GAMT should maintain low levels of GAA, and it has been demonstrated that high GAA levels result in neurotoxicity [70]. Creatine supplementation has been reported to increase brain PCr content by 5–15%, subsequently, brain bioenergetics, and decrease mental fatigue [1]. 

### 5.3. Creatine and Sarcopenia in Advanced Chronic Liver Disease

Sarcopenia is a progressive and generalized skeletal muscle disorder associated with several poor outcomes. The operational definition of sarcopenia by the European Working Group on Sarcopenia in Older People (EWGSOP) has changed over the years, considering as a forefront aspect muscle strength compared to muscle mass because it is recognized that strength is better than mass in predicting adverse outcomes; Specifically, sarcopenia is probable when low muscle strength is detected. The presence of low muscle quantity or quality confirms a sarcopenia diagnosis. When low muscle strength, low muscle quantity/quality and low physical performance are all detected, sarcopenia is considered severe [71].

Sarcopenia is an abnormality in patients with cirrhosis ranging from 40–70%, and it is related to increased complications pre- and post-liver transplantation. In fact, it is associated with a series of negative prognostic elements such as a higher rate of infection, longer hospitalization, hepatic encephalopathy, poor quality of life, and increased healthcare cost. Furthermore, sarcopenia is also associated with poor survival in patients with hepatocellular carcinoma (HCC) [72,73,74,75,76,77,78,79,80,81,82,83,84,85,86,87]. While in these kinds of patients, many data demonstrate the prognostic burden and significance of sarcopenia, there are only a few data on its pathogenic mechanism [72]. Pathogenesis seems to be multifactorial, but the final effect is an important imbalance between protein anabolism synthesis (anabolism) and breakdown (catabolism). For these reasons, considering the clinical importance of sarcopenia, an optimal goal should be to identify pharmacological treatment to slow down sarcopenia and, ideally, to improve muscular mass. We all know that resistance training associated with adequate protein intake is the best treatment to oppose sarcopenia [4,72], but we also know how difficult and full of limits in clinical practice. 

Several factors are involved in regulating muscle mass: cellular energy level, availability of branched-chain amino acid (BCAA), altered endocrine system (e.g., insulin impairment and resistance, IGF-1, corticosteroids, testosterone), cytokines (e.g., TNF alpha), myokine (myostatin), and exercise (no, low, medium or high activity). Moreover, chronic catabolic conditions such as cancer cachexia, increased energy expenditure due to chronic inflammation, reduced food intake due to loss of appetite, and early satiety contribute to muscle catabolism [72]. 

In cirrhosis, as a chronic condition, patients can present most of the previously cited influencing factors such as increased energy expenditure, physical inactivity, decreased food intake with low energy intake (<30 kcal/ ideal body weight) that contribute to the need to deplete body fat and protein stores in order to get energy [72,88,89].

It is also known that a cirrhotic patient has a faster sense of starvation and parallelly cannot adapt correctly to the fast cause of impaired hepatic function. Within ten hours of fasting, fatty acid oxidation and muscle and hepatic glycogen depletion occur in the same measure and way in healthy subjects after three days of starvation. In this setting, muscle and fat catabolism associated with accelerated starvation might serve as a compensatory mechanism to provide glucose for the liver [90]. However, hepatic glycogen storage is limited, and hepatic gluconeogenesis is elevated and guaranteed by lactate and alanine produced by muscle glycogen, protein hydrolysis, and glycerol released from adipose tissue transported to the liver and served as substrates [72].

Although, unfortunately, the mechanism leading to muscle atrophy in cirrhotic patients has not been clearly and definitely identified, hyperammonemia, muscle catabolic autophagy and alterations in endocrinal hormone levels like lower levels of testosterone and BCAA are considered potential contributors to sarcopenia [72].

In particular, hyperammonemia leads to mitochondrial dysfunction that increases reactive oxygen species formation and subsequently oxidative stress, damage and breakdown of muscle protein and lipid; therefore, hyperammonemia decreases muscle protein synthesis by, among other pathway mechanisms, myostatin activation, a myokine [91,92,93,94]. Myokines maintain muscle mass, function, and strength and regulate metabolism in muscles and other tissues and organs, including the liver and adipose tissue. Myostatin is highly expressed in atrophic skeletal muscles and stimulates protein catabolic processes, preventing protein synthesis, muscular hypertrophy, and muscular hyperplasia by inhibiting the activation of satellite cells [95]. Hence, myokines, particularly myostatin, play an important role in regulating muscle metabolism; however, it is unclear whether their production is altered in cirrhotic patients contributing to sarcopenia. Notably, in patients evaluated for LT, there is a significantly higher level of myostatin [72,96,97].

### 5.4. Rational for Creatine Supplementation in Advanced Chronic Liver Disease

**Figure 2:**
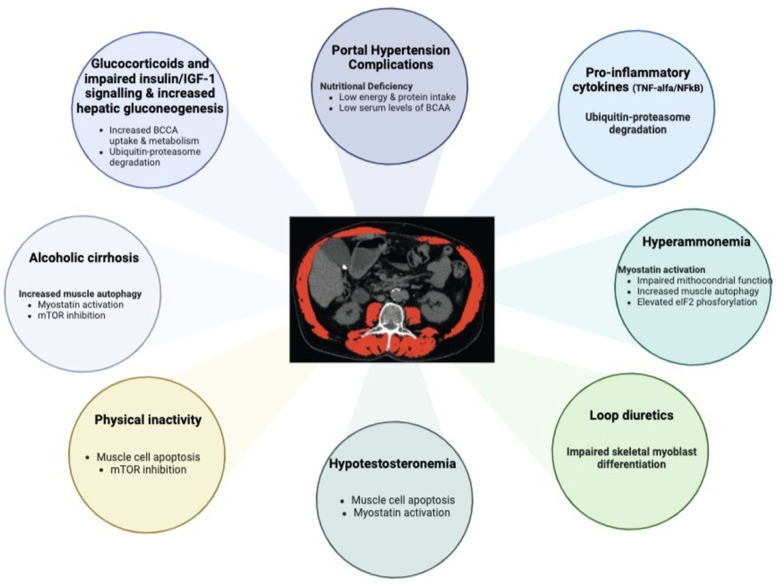
Factors and pathways are influencing sarcopenia in advanced chronic liver disease. Physical inactivity, elevated hepatic gluconeogenesis, impaired insulin/IGF-1 signalling, alcoholic cirrhosis, portal hypertension complications, pro-inflammatory cytokines, hyperammonemia, loop diuretics and hypotestosteronemia are all factors associated with sarcopenia in advanced chronic liver disease. Several signalling pathways, in particular, myostatin activation and ubiquitin–proteasome degradation, influence and alter mitochondrial function; mTOR inhibition, NFkB signalling, apoptosis and elevated eIF2 phosphorylation are involved in sarcopenia in advanced chronic liver disease. BCAA branched-chain amino acids, eIF2 eukaryotic initiation factor 2, IGF-1 insulin-like growth factor 1, NFkB nuclear factor-kB. Modified by [72].

Creatine supplementation in cirrhotic patients could have a role (Figure 2) in treatment depending on several reasons:

First, creatine is a key element in anaerobic energetic cellular metabolism and needs an optimal level. As previously seen creatine synthesis process is mainly realised in the liver, and among all metabolic problems due to a damaged liver, the endogenous creatine synthesis is also compromised [3,65], resulting reasonably at an insufficient level for body needs. 

Second, in cirrhotic patients, muscular mass can shift to sarcopenia. A reduced number of mitochondria or impaired mitochondrial function has been observed in the skeletal muscle of patients with Child–Pugh class B and C cirrhosis [98]. A reduced mitochondrial rate of ATP synthesis in skeletal muscle is associated with energy decrease, delaying anabolic processes such as protein synthesis [72]. Moreover, it seems that creatine can stimulate mitochondrial function, protecting them and the tissue from oxidative damage [99,100,101,102]. Mitochondrion characterises aerobic energetic cellular metabolism, contrary to anaerobic glycolysis and creatine characterising anaerobic energetic cellular metabolism. These three mechanisms do not work separately but together [7]. This means that with diminishing mitochondrial concentration and function in the muscular cell of the cirrhotic patient, energetic cellular metabolism could require more than normal from the anaerobic metabolism, usually utilised for energetic needs of a very short time [7]. Therefore, creatine could be an important energy resource in cirrhotic disease, where we find limited hepatic glycogen content [72]. 

Third, clinical data widely demonstrated how creatine, even in the elderly population associated with resistance training, can improve performance and muscular mass [9]. Lastly, creatine is involved in different molecular pathways, particularly in inhibiting myostatin [103], a myokine that is well-known to limit muscular development [72]. Thus as previously seen, creatine has an important role in energetic cellular metabolism. Its endogenous synthesis in the liver is about 50% of the daily requirement, whereas the other 50% comes from the diet [1,7,8,9,14]. The liver plays a key role in creatine production, suggesting creatine could be negatively influenced by liver diseases [3]. 

Therefore, a chronic disease condition as occurs in chronic liver disease/cirrhosis causes an important energetic consumption with the need to take energy by the three energetic substrates [72]: carbohydrates, lipids and proteins. The first and main used substratum is glucose (carbohydrate) which is present in many supplies in the shape of glycogen in the liver [7]. This one is unfortunately limited in the cirrhotic liver [72], maybe because of fast and ravenous use to produce energy. This lead to the need to increase gluconeogenesis by lipidic and protein catabolism. This occurs in many tissues, particularly in muscular ones greedy for glucose [7,72]. 

Creatine is an osmotically active substance known as a retention substance, driving water intracellularly, particularly in muscular cells where is present 95% of body creatine; although creatine supplementation increases total body water, fluid distribution is not altered [23]. Interestingly, an increase in cell volume appears to be an anabolic proliferative signal, which is supposed to be the first step in mouse protein muscular synthesis [104,105]. In this context, creatine supplementation could represent a new strategy to improve some of the conditions characterising cirrhotic patients starting from sarcopenia but also in encephalopathy linked to hyperammonaemia, fatigue and, maybe, ascites.

Creatine supplementation would help to balance the loss of production in liver disease leading to an optimal concentration in muscle and improving sarcopenia by the series of mechanisms seen; therefore, it could have a neurological protective role against hyperammonaemia and improve physical and cognitive fatigue [1,106,107,108]. 

Interestingly the catalysis of the ATP reservoir system, Cr/PCr, by creatine kinase (CK) is not present in the liver because it is not expressed in hepatocytes. Although the liver represents the final step of creatine synthesis, it has little cytosolic CK, mainly expressed in muscles, the brain, and the heart. An elegant experiment showed how the induced expression of CK with abundant phosphocreatine in transgenic mice after hepatectomy could improve liver regeneration by refilling hepatic energy metabolism [109]. Maintaining an optimal ATP level in hepatic cells also could protect from sepsis-induced liver injury and mortality, as was shown in creatine kinase transgenic mice fed with creatine supplementation [110]. 

To our knowledge, no study in humans has evaluated creatine supplementation in cirrhotic patients, and particularly no one has evaluated the effect of creatine supplementation on sarcopenia, fatigue, encephalopathy and, in general, improving chronic liver disease.

## 6. Future Perspective of Creatine in Liver Diseases

As we have seen, creatine is a fundamental product of the liver, which reasonably, in disease conditions, cannot guarantee an adequate endogenous level of creatine. We also have seen how much creatine is important in several metabolic processes and linked to several disease conditions. For these reasons, creatine supplementation could benefit patients with chronic liver disease. First, in patients with NAFLD/NASH, as shown in the animal model, creatine supplementation may treat and prevent lipid accumulation in the liver. Also, for cognitive impairment in cirrhotic patients, particularly in encephalopathy, creatine could have a neuroprotective role in preventing and contrasting hyperammonaemia effects on the brain, as shown in other disease conditions.

Moreover, fatigue (physical and cognitive), a debilitating symptom in patients affected by chronic diseases, could benefit from creatine supplementation. Not only creatine has the property of intracellularly driving water, particularly in muscular cells: a long shot, it can be hypothesised a role of creatine in helping to prevent fluid decompensation. Lastly but principally, the more interesting and surely promising aspect is the role of creatine supplementation on sarcopenia to prevent and treat this important condition associated with poor outcomes and prognosis, such as it already occurs for the elderly subject who is administered with creatine.

Ideally, creatine supplementation in patients with the chronic liver disease could act on several critical points of this condition and its complications. Further studies are needed to support the clinical burden of this hypothesis in chronic liver disease.

## Data Availability

Not applicable.
